# Structural and hemodynamic analysis of Weaire-Phelan scaffolds made of Ti-alloy as bone replacement component: A preclinical investigation

**DOI:** 10.1371/journal.pone.0312880

**Published:** 2024-12-02

**Authors:** Jaideep Singh Bhardwaj, Souptick Chanda

**Affiliations:** Department of Biosciences and Bioengineering, Indian Institute of Technology Guwahati, Guwahati, Assam, India; Khalifa University of Science and Technology, UNITED ARAB EMIRATES

## Abstract

In recent years, additively manufactured metallic scaffolds have generated significant interest among researchers working in the field of bone tissue engineering and orthopaedic implants. Although such intricate, porous architectures are promising as bone substitutes, they need to be thoroughly tested for structural robustness as well as their capacity for bony integration. In this present work, we introduced and preclinically evaluated the biomechanical viability of Weaire-Phelan (WP) Ti-alloy scaffolds as bone replacement components. Two distinct groups of WP scaffolds, namely WPA and WPD, of varying porosities were examined for comparative assessment. Finite element (FE) analysis, computational fluid dynamics (CFD) and uniaxial compression tests were performed on 3D printed as-built scaffolds to comprehensively evaluate the structural, hemodynamic, fatigue and morphometric properties of the two groups. The mechanical performances of the WP scaffolds of 70%, 80% 90% porous group (relative density 0.3 and lower) were found to accord with the natural trabecular bone tissue. However, WPA scaffolds demonstrated slightly superior mechanical performances as compared to WPD scaffolds (22%– 63% greater compressive modulus depending on the porosity). On the other hand, WPD scaffolds showed improved hemodynamic properties thereby implying enhanced osteogenic potential. Moreover, the range of effective elastic moduli corresponding to the WP scaffolds was found to be in good agreement with that of the natural bone tissue. As such, these designs were categorized based on their suitability at different anatomical sites. The overall performance metrics of the WP scaffolds underscore its potential for improved osseointegration, structural conformities and greater capacity for customization with enhanced manufacturability.

## 1. Introduction

In the domain of bone tissue engineering, the focus on creating porous metallic bone scaffolds or metamaterials using additive manufacturing techniques, e.g., selective laser melting (SLM), has intensified lately. These metallic structures provide enhanced mechanical strength, making them appropriate choice for applications that require the ability to support body weight. Among metals, titanium alloys (e.g. Ti6Al4V or Ti64) are clinically favoured for scaffolding applications. Apart from being a value-optimised proposition, Ti64 has exceptional biocompatibility, mechanical strength, low weight and high anticorrosive properties for various medical applications [1–5]. However, such additively manufactured metal scaffolds for bone applications need to be evaluated for numerous physical characteristics and subsequently optimized during the scaffold designing stages. It is worth noting that several critical parameters, such as elastic modulus, relative density, permeability, wall shear stress (***WSS***), porosity, and pore size, may impact both the mechanical and biological efficacy of the scaffold [6–12].

To ensure stable support and minimize negative effects of stress shielding, it is crucial that the bone scaffolds’ effective elastic modulus (or stiffness) is comparable in situ with the host bone tissue [13, 14]. This value typically ranges between 0.01 to 18 GPa [15]. The permeability of the bone scaffold should also be comparable (the recommended value lies in the range 0.5×10−8–5.0×10^−8^ m^2^) [16–20]. Permeability levels that fall below the designated threshold may lead to obstructions, thereby hindering the efficient exchange of waste and nutrients through the implanted scaffold [21]. On the other hand, permeabilities significantly greater than the optimal range are unfavourable too, since they result in cell washout [22]. While conceiving the scaffolds design, it is essential to consider the porosity and pore size of unit cells as they are the other key physical attributes of bone scaffolds. Porosities in the range 70% to 90% [23–25], and pore sizes in the midst of 400–1000 μm [26–29] are ideally suited for bone regeneration. ***WSS*** also plays a major role since its value over the 60 mPa causes apoptosis [30]. One way to meet these mechanical and biological requirements is to modify the internal architecture of the scaffold’s unit cells during the design steps. Hence, both structural and hemodynamic assessment of scaffolds are critical before contemplating any clinical trial.

Ever since the beginning of this millennium, numerous studies have explored different porous structures for possible use in orthopaedic applications [31–40]. Based on their architectures, engineered scaffolds were broadly categorized into two types: (A) strut-based scaffolds, also known as skeletal type and (B) surface-based scaffolds, also known as triply periodic minimal surface (TPMS) type. Strut-based scaffolds can further be categorised based on their unit cells architecture, e.g. cubic, body centred cubic (BCC), face centred cubic (FCC), honeycomb, Voronoi and diamond structure. Similarly, TPMS type can be categorised as: Gyroid, Schwarz, Split-P and I-WP [41]. TPMS scaffolds were reported to have enhanced mechanical characteristics in comparison to skeletal type scaffolds; however, skeletal scaffolds prevail better in fluid-flow (or hemodynamic) operations [42–45]. Despite various scaffold patterns were reported lately, they were not investigated on the basis of both mechanical and biological aspects as criteria in bone application. Therefore, current bone scaffolds–degradable and non-degradable–still face issues, such as stress shielding, interfacial loosening and early failure.

**Fig 1:**
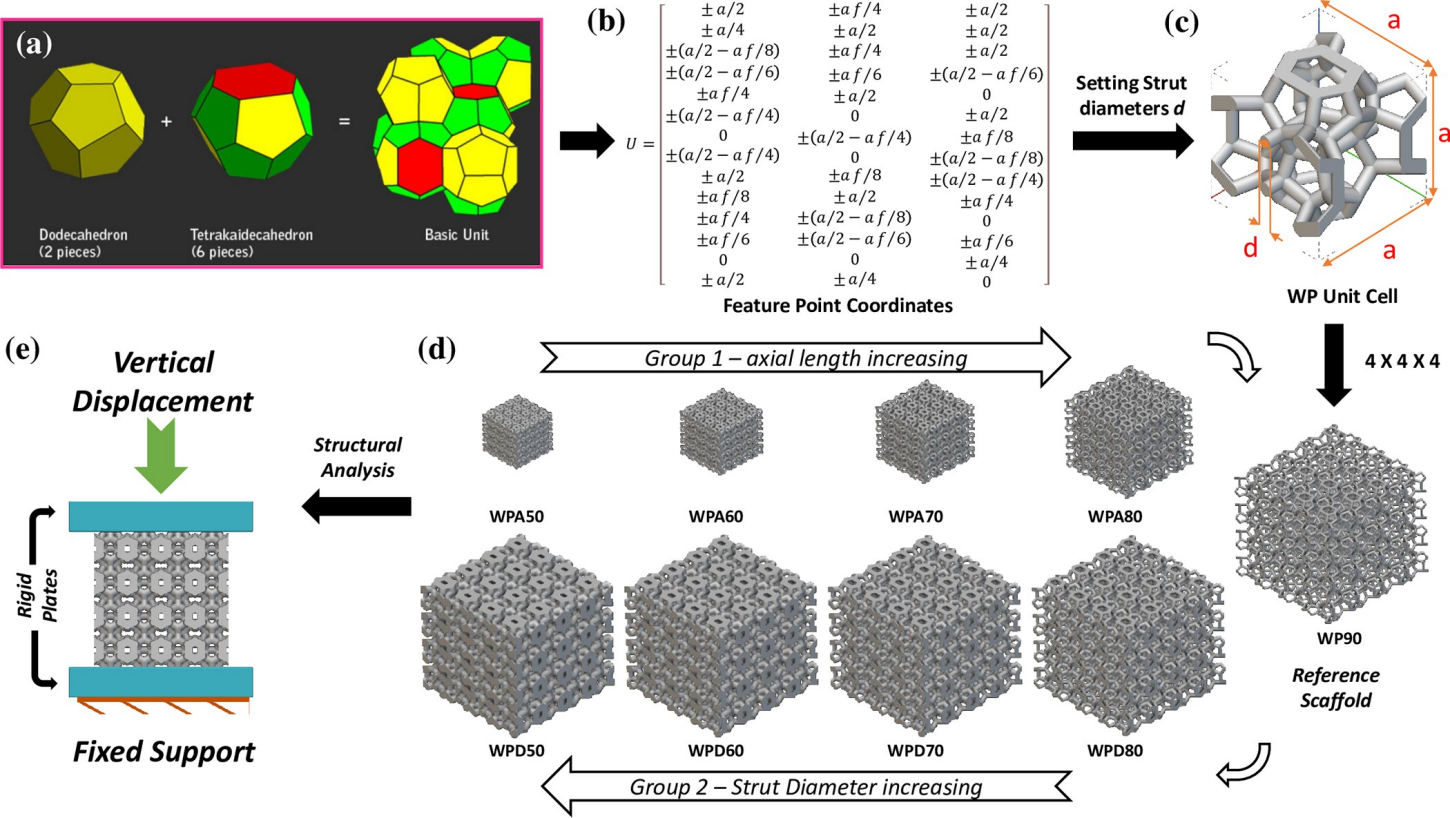
3D Scaffold modelling for FE analysis: (a) two sub-units of basic unit cells in WP structure; (b) feature point coordinates for 3D modeling; (c) WP scaffold unit cell; (d) two groups of scaffold models along with reference scaffold and (e)the boundary condition applied in FE analysis.

Weaire-Phelan (WP) structure was discovered in 1994 by D. Weaire & R. Phelan as unit cells with lower surface area per unit volume (by about 0.3% less than Kelvin cell, 1987). The WP unit cell shown in Fig 1A is made up of six 14 sided cells (tetrakaidecahedron: with 12 pentagonal & 2 hexagonal faces) and two 12 sided cells (pyritohedron:12 irregular pentagonal faces), all equal volume. The tetrakaidecahedron are organized in three orthogonal axes with the pyritohedron staying in the interstices amongst them, giving an overall simple cubic interconnected structure [15, 46]. Although promising, these scaffolds were not extensively studied for their suitability in bone applications. While some structures fulfilled mechanical requirements, the others were only tested for biological requirements. A thorough comparison based on in vitro and in silico performances of the WP scaffolds with customized architectural features may result in an improved alternative to lattice-based scaffolds for greater success rate in bone applications.

In this study, nine different WP scaffolds were investigated on the basis of structural and hemodynamic performance. A WP scaffold with a porosity of 90% was selected as the reference model. Eight additional scaffolds were designed with their architectural features adjusted based on the reference scaffold. These eight scaffolds were divided into two groups, Group-1 and Group-2, each containing scaffolds with different levels of porosity: 50%, 60%, 70%, and 80%. Afterwards, the designed scaffolds were numerically evaluated for compressive modulus (***E***_***c***_), yield strength (***YS***), fluid flow pattern, pressure drop across length, permeability and ***WSS***. Furthermore, the numerical estimations were validated by executing axial compression test on additively fabricated scaffolds, and thereafter comparing with existing material models for bone application.

## 2. Materials and methods

### 2.1 Scaffold design

Applying WP structure as scaffolds’ unit cell, nine cubic scaffold models were designed using nTopology^®^ (nTopology^®^, v3.40.2, New York USA) software as shown in Fig 1A–1D. The WP structure’s unit cell scaffold was constructed by altering the lattice WP structure to achieve triaxial symmetry and improved modeling adaptability. A parametric design approach for the WP structure is depicted in Fig 1B and 1C, which incorporates three geometric parameters: the axial length (or the bounding length of the unit cell) denoted as ***a***, the strut diameter represented as ***d***, and the shape control coefficient symbolized as ***f***. The shape control coefficient ***f*** is determined by the perpendicular distance from the pyritohedron’s mass centre to the tetrakaidecahedron’s contact face, denoted as ***L***_***0***_, and the total distance between the mass centres of the neighbouring tetrakaidecahedron and pyritohedron, represented as ***L***. The shape control coefficient ***f*** is thus defined as the ratio of ***L***_***0***_ to half of ***L***, i.e., ***L***_***0***_***/(L/2)***, the value of which typically ranges from 1.0 to 1.5 [16, 46].

A WP scaffold unit cell with a porosity of 90% (WP90) was created as the reference model. Eight additional scaffolds were designed by adjusting the architectural features of W90. These eight scaffold models were divided into two study groups (Group 1 and 2) based on the difference in their unit’s axial length (***a***) or strut diameter (***d***) (Fig 1C and 1D). In Group 1 type, the length of the struts (i.e. axial length) was varied and the strut diameter was kept constant. As such, the Group 1 scaffolds were named WPA (Weaire-Phelan—variable axial length). Similarly, the Group 2 scaffolds were named WPD (Weaire-Phelan—variable strut diameter) since the strut diameter was varied while strut length was kept constant to control the porosity. Each group contained four scaffolds with different levels of porosity: 50%, 60%, 70%, and 80%. The four scaffolds in Group 1 with unique relative densities (or porosities) were generated by reducing the axial length of the reference unit, resulting in creation of WPA50, WPA60, WPA70 and WPA80, where the number denotes percentage of porosity. Similarly, the other four scaffolds in Group 2 having similar relative density as in Group 1 scaffolds were created by increasing the strut diameter of the reference unit, resulting in the creation of WPD50, WPD60, WPD70 and WPD80 models. The feature details of the designed scaffolds are listed in Table 1.

**Table 1:** Structural features and dimensional properties of designed scaffold models.

	Scaffold name	Unit cell		Scaffold (CAD)		
		Axial length, *a* (mm)	Strut diameter, *d* (mm)	Bounding volume, *l*×*l*×*l* (mm^3^)	Estimated porosity, *P* (%)	Estimated relative density
**Group 1**	WPA-50	1.12	0.25	4.4×4.4×4.4	48.59	0.51
	WPA-60	1.3	0.25	5.2×5.2×5.2	59.87	0.40
	WPA-70	1.55	0.25	6.2×6.2×6.2	69.68	0.30
	WPA-80	2.0	0.25	8×8×8	80.31	0.20
**Group 2**	WPD-50	3.0	0.68	12×12×12	48.83	0.51
	WPD-60	3.0	0.58	12×12×12	59.53	0.40
	WPD-70	3.0	0.48	12×12×12	70.05	0.30
	WPD-80	3.0	0.37	12×12×12	80.48	0.20
**Reference model**	WP-90	3.0	0.25	12×12×12	90.49	0.10

The units of each scaffold were arrayed in a 4×4×4 pattern to generate the final 3D model of these scaffolds. Fig 1D presents isometric views of the two groups of scaffold cubes, whereas the morphometric parameters of the unit and the scaffold, such as the axial length ***a*,** strut diameter ***d*,** bounding volume ***V***_**0**_, estimated porosity ***P*** and relative density $\frac{\rho^{*}}{\rho_{0}}$ are listed in Table 1. Theoretical ***P*** and $\frac{\rho^{*}}{\rho_{0}}$ estimation methods are defined in Eq 1 and 2 as follows:

$$P=\frac{V^{*}}{V_{0}}\times 100\%$$ (1)

$$\frac{\rho^{*}}{\rho_{0}}=1-\frac{P}{100}$$ (2)

where V* represents the total theoretical pore volume of the scaffold.

### 2.2 Fabrication and imaging of the scaffolds

Direct metal printing (DMP) technology was used for fabricating the scaffolds [11, 12]. DMP—a special type of SLM—is a highly promising additive manufacturing technique. Ti6Al4V powder (LaserForm^®^ Ti Gr23(A) Alloy powder, 3D Systems, USA) was used to fabricate the scaffolds employing a DMP system (DMP Flex 350 Printer, 3D Systems, USA), which is fitted with a 500W Fiber laser. Pre-optimised process parameters of the machine as listed in Table 2 were used to attain maximum built quality.

**Table 2:** SLM process parameters.

Laser power (W)	Laser scanning Speed (mm/s)	Laser Diameter (μm)	Scanning Angle (°)	Layer thickness (μm)	Hatch spacing (μm)	Gas atmosphere
125	700	55	245	30	110	Argon

In order to increase ductility, standard heat treatments for Ti-alloys were carried out inside inert Argon gas chamber for stress relieving. Fabricated scaffolds were heat treated at 850°C (± 10°C) for 120 min (± 30 min) while a constant flowrate of Argon was maintained at 500 ltr/hr. This was followed by subsequent cooling (i.e. Argon quenching) for 720 minutes. Although mechanical properties are relatively insensitive to changes in heating and cooling rate, longer treatment time may result in decreased strength and increased elongation [47]. The assessment of surface morphology and geometrical characteristics was conducted using an optical microscope (Leica S9 Digital Stereozoom microscope, Germany). The microscopic image processing and micro-architectural measurements were performed on ImageJ software (ImageJ^®^ 1.54e, NIH, USA).

### 2.3 In vitro ductility tests

**Fig 2:**
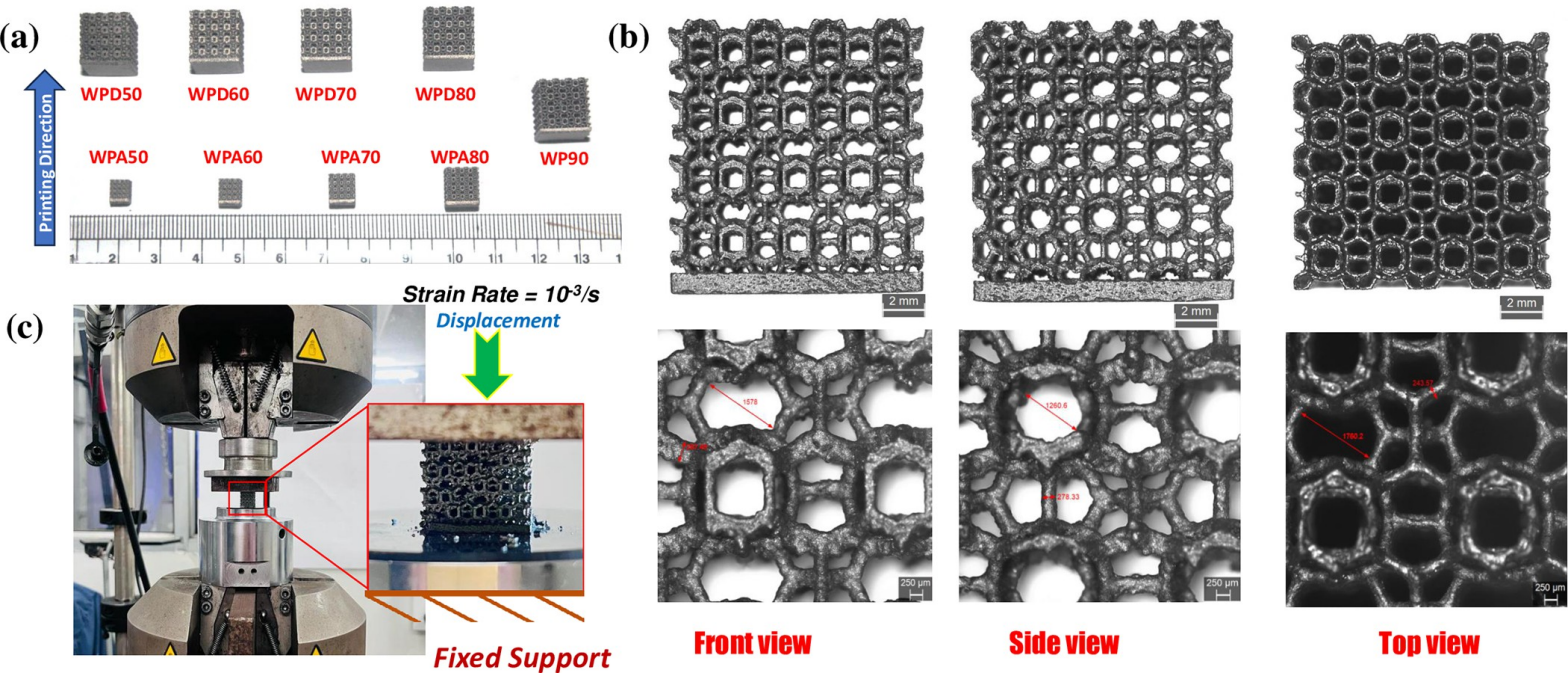
Uniaxial compression test: (a) additively manufactured Ti64 metal WP-scaffold models; (b) digital microscope images of a scaffold from front, side and top views enlarged at 1X (up) and 3X (down) and (c) uniaxial compression test setup at 250kN UTM.

Uniaxial compression tests on fabricated scaffolds (Fig 2A) were performed using universal testing machine (UTM) (BISS^®^, Median servo hydraulic UTM, ITW India) with a 250 kN load cell as shown in Fig 2C. Fabricated samples were placed centrally in between two parallel plates of the UTM one by one, so that the loading axis aligns vertically with the centre of the sample. Bottom plate was fixed and the top plate gradually moves downwards to apply the load. Samples were compressed in the additive layering direction with a constant crosshead speed at initial compression strain rate of 10^−3^ s^-1^ to ascertain a quasi-static loading following ISO standard (ISO 13314:2011) [48, 49]. The compression tests were terminated when displacement reached ~60% or more of scaffold height. The test pictures were captured with a digital camera (Canon EOS 3000D). The tests were conducted on three samples of each set. The engineering stress-strain data were recorded, analyzed and averaged in OriginPro^®^ software (OriginLab Corporation, SR1, Massachusetts, USA) and the average curves were plotted. Standard deviations of the average data points were obtained from statistical analysis. The mechanical properties including ***E***_***c***_ and ***YS*** were calculated from the final curves of each set. Apparent ***YS*** values were estimated based on ‘0.2%-offset strain’ method.

### 2.4 Structural analysis using FE models

Quasi static compression tests on scaffold’s FE models were simulated in Ansys workbench (Ansys^®^ Mechanical, Release 2022 R2). High-quality solid mesh applying non-linear 10-noded tetrahedral elements (C3D10) was generated using Ansys® meshing tools. Mesh size was adjusted through a mesh sensitivity process to strike a balance over precision and computational speed. The resulting elements size was 1/8 to 1/4 of strut diameter. The element quality ranged from 0.7 to 0.9, with a Jacobian ratio between 1 and 10, an average skewness from 0.1 to 0.4 and orthogonality scores spanning from 0.85 to 1.0 across all scaffolds examined. Multilinear plasticity hardening (elastoplastic) material model of Ti64 was obtained from tensile test of additively manufactured dog-bone structure [50] and employed for FE analysis (elastic modulus = 91,621 MPa and Poisson’s ratio = 0.33, yield strength = 1080 MPa, ultimate strength = 1368 MPa). Two rigid plates were placed on the top and bottom surface of the scaffold as shown in Fig 1E. Bottom plate was fully constrained for all six degrees of freedom, while the top plate was limited to axial movements. The top plate was displaced axially, resulting in the scaffolds being compressed to 25% of their original height. The scaffolds were compressed in the ***z***-direction. The auto time step feature in Ansys® was used, allowing each simulation round to be completed in 27–100 time steps. the reaction force extracted from the top plate in FE simulations was used to predict the compressive modulus, ***E***_***c***_, of scaffolds employing the Eq 3. Stress vs. strain curves were obtained from non-liner FE analysis results.

$$E_{c}=\frac{\sigma }{\varepsilon };\;\sigma =\frac{F}{A_{0}};\;\varepsilon =\frac{\Delta l}{l}$$ (3)

where ***σ*** is compression stress (MPa), ***ε*** is strain (mm/mm), ***F*** is reaction force (N), and ***A***_**0**_ is cross-sectional surface area vertical to the compression axis (mm^2^), **Δ*l*** is directional deformation of the scaffold and ***l*** is the initial height (mm) of the scaffold.

### 2.5 Prediction of mechanical properties

The Gibson-Ashby (G-A) model is frequently used to examine and forecast the impact of relative density (i.e. volume fraction) on the corresponding modulus of elasticity and ***YS*** for open cellular structures, such as cellular solids and scaffolds [15, 46, 51]. The relationship between relative density (ρ*/***ρ***_**0**_) and relative modulus of elasticity (E*/***E***_**0**_) can be expressed as per Eq 4:

$$\frac{E^{*}}{E_{0}}={C(\frac{\rho^{*}}{\rho_{0}})}^{n}$$ (4)

where E^*^, ρ* are the modulus of elasticity and apparent density of the scaffold, respectively, whereas ***E***_**0**_,***ρ***_**0**_ represent the modulus of elasticity and density, respectively, of bulk material of which the scaffold is made up. ***C*,*n*** are constants linked with porous material, derived by fitting the experimental outcomes based on Eq 4. For open cellular structures, the range of predicted values of ***C*** = 0.1–4, while ***n*** = 1 for stretching controlled rod and plate-like microstructures, ***n*** = 2 for bending or twisting controlled rod like microstructures and ***n*** = 3 for bending controlled plate-like microstructures [15, 41].

Carter and Hayes [52] characterized the modulus of elasticity of trabecular bone, ***E***^***T***^, as being non-linearly related to its bulk density, ***ρ***_**0**_. The relationship between ***ρ***_**0**_ and ***E***^***T***^ can be expressed as per Eq 5:

$$E^{T}=3790\;\epsilon^{0.06}\;\rho_{0}^{3}$$ (5)

where ***ϵ*** is strain rate per second. The most remarkable aspect of this power law relationship is its applicability across the full spectrum of skeletal bone apparent density, i.e. from dense cortical bone to the highly porous trabecular bone. Therefore, mechanical test results of this study were compared with the bone’s mechanical properties using the power law relation as described in Eq 5.

### 2.6 Hemodynamic analysis of the scaffolds

**Fig 3:**
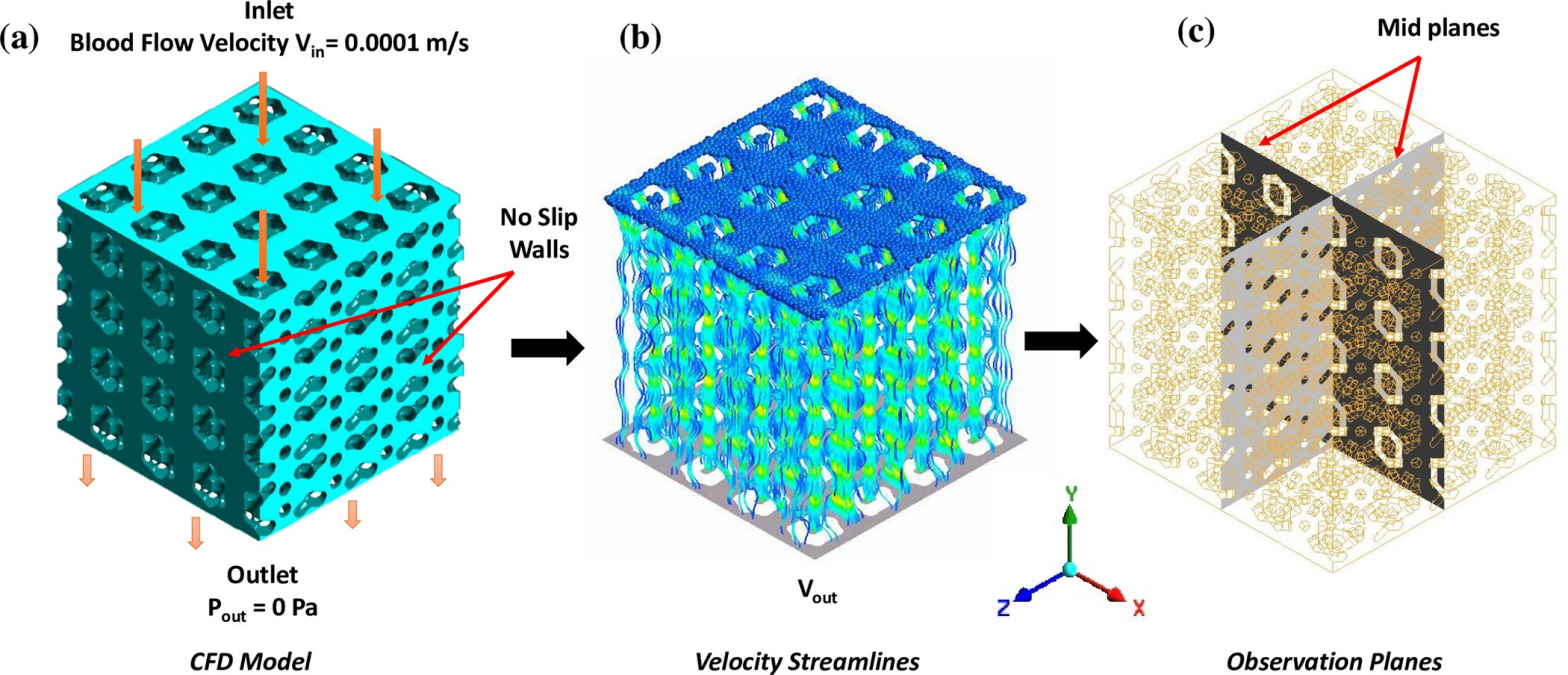
Hemodynamic analysis: (a) extracted porous domain of scaffold for CFD analysis; (b) velocity contours through the porous domain of scaffold and (c) cross-sectional planes of porous domain where velocities are depicted.

Blood flow on the porous domain of the scaffolds was simulated in Ansys workbench (Ansys^®^ CFX, Release 2019 R2) using finite volume method (FVM). Porous domain or void volume of the scaffolds was extracted from bounding volume and considered as fluid domain (Fig 3A). FE models of the fluid domain of the scaffolds were prepared using nTopology^®^ software and subsequently exported to Ansys.

High-quality solid mesh applying linear tetrahedral elements (C3D4) was generated using nTopology® meshing tools. The mesh size was fine-tuned through a mesh sensitivity process to strike a balance between precision and computational speed. The resulting elements size was 1/8 to 1/4 of smallest features. The element quality ranged between 0.8 to 0.9, an average skewness from 0 to 0.3 and orthogonality scores spanning from 0.85 to 1 across all scaffolds examined. Blood flow was characterized (see Table 3) by flow of an incompressible Newtonian fluid having dynamic viscosity, ***μ***_***d***_ = 0.0045 Pa.s, inlet velocity, ***v***_***i***_ = 0.0001 m/s, medium turbulence (5%), and zero outlet pressure, i.e. ***p***_***o***_ = 0 Pa in ***z***-direction [53–56] (Fig 3). Rigid walls with no-slip boundary conditions were assigned on the lateral surfaces and a steady state flow was modelled to effectuate the pressure drop across the porous domain (Fig 3B). The root-mean-square (rms) value of 1×10^−4^ was set as the convergence standard for the residuals [57, 58]. In order to replicate physical conditions of typical human body, we used isothermal heat transfer at 37°C and a shear stress transport (SST) turbulence model. The SST model, a dual-equation ***k-ω*** model that enhances the forecast of separation close to the walls of the model [58, 59]. Such models can accommodate for turbulence, a physiological trait of blood flow [60, 61].

**Table 3:** Hemodynamic input parameters for computational fluid flow analysis.

Molar Mass (kg/kmol)	Density (kg/m^3^)	Heat capacity (J/kg.K)	Dynamic Viscosity (Pa.s)	Heat Conductivity (W/m.K)
65000	1050	4000	0.0045	0.45

The conservation of mass and momentum throughout the porous domain of the scaffold was addressed by employing the 3D Navier-Stokes equations using Finite Volume Method (FVM). The equations that govern the flow of an incompressible viscous fluid are presented in Eq 6 and 7 [59] as described below:

$$\rho \frac{\partial u}{\partial t}-\mu_{d}\nabla^{2}u+\rho (u.\nabla )u+\nabla p=F$$ (6)

∇.u=0 (7)

where ***ρ*** is fluid density (kg/m^3^), ***t*** is time (s), ***u*** is fluid velocity (m/s), ***μ***_***d***_ is dynamic viscosity (Pa. s), ***p*** is pressure (Pa) and ***F*** denotes other forces (i.e., gravity or centrifugal force) acting in the fluid domain. In this study, ***F*** is considered zero since gravity and other forces were assumed to have negligible effect on the fluid domain.

The permeability of the scaffolds, was calculated according to Darcy’s law as described in the Eq 8 [54, 62, 63]:

$$k=v_{d}\mu_{d}(l/\Delta p)$$ (8)

where, ***k*** is permeability of scaffold (m^2^), ***v***_***d***_ is Darcy’s velocity or superficial outlet velocity, ***l*** is length of the porous medium and ***Δp*** is average pressure drop across the porous domain.

By taking into consideration the normal velocity gradient over a filament and that each scaffold operates under a laminar flow regime, the ***WSS*** can be computed as per Eq 9 [61, 62, 64–67]:

$$\tau_{w}=\mu_{d}\frac{\partial u}{\partial n}$$ (9)

where ***τ***_***w***_ is the ***WSS*** (Pa) and ***∂u***/***∂n*** denotes velocity gradient in ***x*, *y***, and ***z***-directions. The raw values for velocity, pressure, and ***WSS*** were extracted through post-processing of the results using the CFX-CFD post suite.

### 2.7 Fatigue analysis

The fatigue factor of safety was determined using the Soderberg method, as illustrated in Eqs 10–13 [68–70]. In the calculation, the minimum, ***σ***_***min***_ and maximum, ***σ***_***max***_ stresses were calculated at loads corresponding to a stress ratio of 0.1×*S*_*ys*_ (R = 0.1) and up to 0.8×*S*_*ys*_ of scaffold.


$$\sigma_{m}=\frac{\left(\sigma_{max}+\sigma_{min}\right)}{2}$$ (10)

$$\sigma_{a}=\frac{\left(\sigma_{max}-\sigma_{min}\right)}{2}$$ (11)

$$\frac{\sigma_{a}}{S_{e}}+\frac{\sigma_{m}}{S_{ys}}=\frac{1}{N}$$ (12)

Hence, the fatigue factor of safety, *N*_*F*_ is as follows:

$$N_{F}=\frac{1}{\frac{\sigma_{a}}{S_{e}}+\frac{\sigma_{m}}{S_{ys}}}$$ (13)

where ***σ***_***m***_ and ***σ***_***a***_ represent the mean and alternating stresses generated in the scaffolds. *S*_*e*_ and *S*_*ys*_ represent the endurance limit of scaffold calculated using Eq 14, and the yield strength of corresponding scaffold, respectively.


$$S_{e}=K_{a}\times K_{b}\times K_{c}\times K_{d}\times K_{e}\times K_{f}\times S_{u}=K\times S_{u}=0.54\times S_{u}$$ (14)

$K_{a}:\;Surface\;quality\;factor$

$K_{b}:\;Size\;factor$

$K_{c}:\;Reliability\;factor$

$K_{d}:\;Temperature\;factor$

$K_{e}:\;Stress\;concentration\;factor$

$K_{f}:\;Miscellaneous\;factor$ where ***K*** represents fatigue strength reduction factor (***K*** = 0.54 for Ti6Al4V [69]) and ***S***_***u***_, the ultimate compression stress of the respective scaffold.

While the number of cycles to finite life, ***N***_***f***_ was calculated using the Basquin Eq 15 and fatigue strength coefficient ***σ***′_***f***_ for each scaffold was determined using Eq 16 at *N*_*f*_ = 1:

$$\sigma_{a}={\sigma \prime }_{f}\cdot {\left({2N}_{f}\right)}^{b}$$ (15)

$${\sigma^{\prime }}_{f}=\frac{\sigma_{a}}{2^{-0.095}}$$ (16)

where ***b*** represents fatigue strength exponent (***b*** = −0.095 for Ti6Al4V [70]). The obtained results were compared with that of cortical and cancellous for fatigue where ***σ***′_***f***_ ***= 26*.*4*, *b = −0*.*155*** for cancellous bones [71], and ***σ***′_***f***_ **= *131*.*7*, *b = −0*.*11*** for cortical bones [72].

## 3. Results

### 3.1 Imaging and ductility tests

Fig 2A and 2B show the fabricated scaffolds and the obtained optical microscopic images. The edges and cross-hatching have been precisely melted and formed, exhibiting no gaps or voids. However, for all scaffold types, some partial melting has been noticed. It was observed from the enlarged images (3X) that some partially melted particles adhered to the scaffold’s exterior surface contributing to somewhat coarse texture, as seen in Fig 2B. Table 4 enlists a summary of feature size and mass fidelity of fabricated scaffold compared with its CAD design, respectively. Despite sporadically present rough surfaces, WPD parts were printed with acceptable consistency and geometrical precision. The greatest discrepancy in geometrical measurement was observed in WP90 scaffold (accounting for ~2.1% of the strut diameter) whereas maximum deviation in mass accuracy (~41.2%) was found in WPA60 scaffold.

**Table 4:** Features size and mass fidelity of fabricated scaffolds over respective scaffold design.

Scaffold archit-ecture	Strut diameter (μm)			Mass (g)			Relative density
	Designed	Measured*	Discrepancy (%)	Designed	Measured**	Discrepancy*** (%)	Calculated
**WPA50**	250	252.09±1.3	0.83	0.194	0.269±0.003	38.7	0.71
**WPA60**	250	250.60±1.5	0.24	0.250	0.353±0.014	41.2	0.57
**WPA70**	250	249.07±1.3	0.37	0.320	0.441±0.015	37.8	0.42
**WPA80**	250	253.81±1.0	1.52	0.446	0.578±0.008	29.4	0.25
**WPD50**	680	679.01±0.9	0.14	3.917	3.535±0.069	9.8	0.46
**WPD60**	580	580.95±1.0	0.16	3.097	2.824±0.008	8.8	0.37
**WPD70**	480	480.88±0.8	0.183	2.292	2.145±0.005	6.44	0.28
**WPD80**	370	372.97±2.2	0.80	1.471	1.464±0.043	0.48	0.19
**WP90**	250	255.39±2.0	2.15	0.727	0.836±0.006	14.87	0.11

*Measured size = *mean* ± *standard deviation*

**Measured mass = *mean*±*range*

***Discrepancy = |*measured−designed*|/*designed*

**Fig 4:**
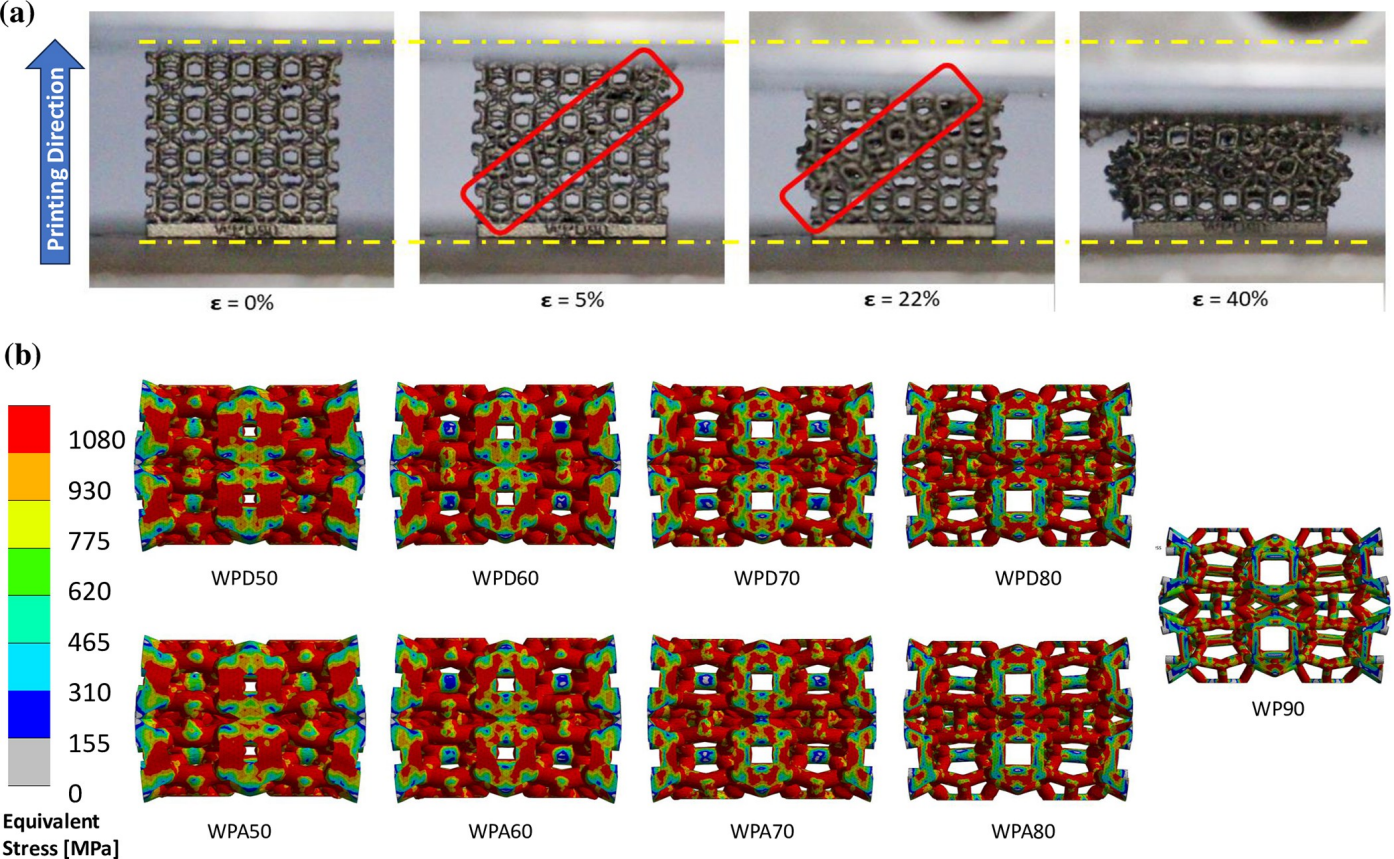
(a) Deformation response of specimen WP90 compressed under UTM machine and (b) equivalent (von-Mises) stress distribution on WP scaffolds as a result of FE analysis.

**Fig 5:**
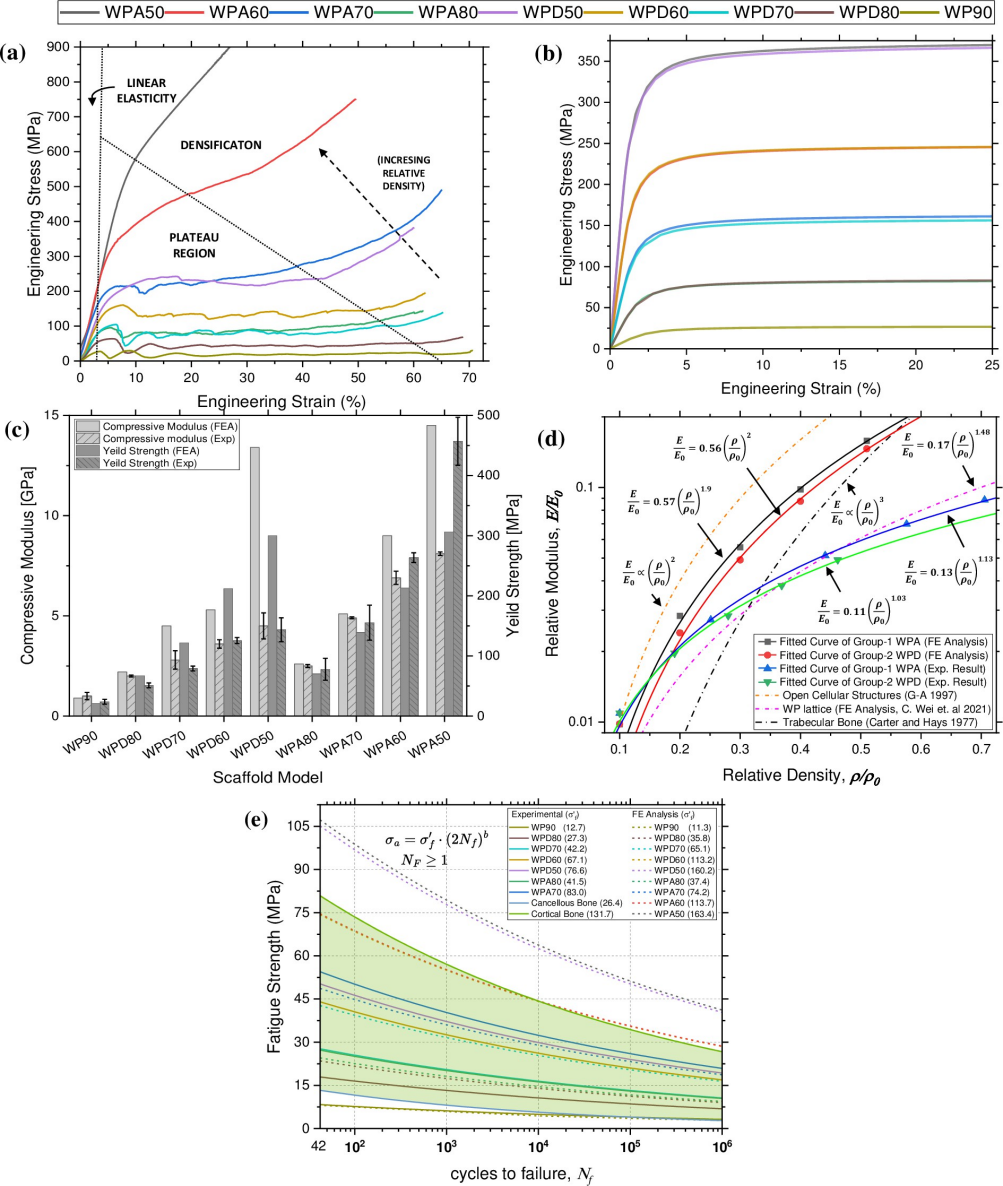
Stress-strain curves relating to various scaffold models from axial compression experiments (a) under UTM; (b) elastoplastic FE analysis and (c) obtained compressive modulus and yield strength of the scaffold models. (d) The relative compressive modulus of WP scaffolds plotted against relative density and compared with existing studies of (i) open cellular structures, (ii) trabecular bone and (iii) FE analysis results applied with Johnson-Cook material model on WP lattices. (e) Fatigue strength to finite life curves of fabricated and designed scaffolds.

Fig 4A shows the observed deformation response of the fabricated WP scaffolds under axial compression and the obtained stress-strain plot is presented in Fig 5A. For every scaffold type, the early portion of the stress-strain graph exhibited a linear trend, which then transitioned into a non-linear pattern after approximately 2% of engineering strain., This was followed by long plateau region arising from cell collapse by yielding, buckling or crushing of struts, thereby allowing large energy absorption at near contact load, truncated by densification and cells touching. Moreover, in intergroup comparison, WPA scaffolds demonstrated greater ***E***_***c***_ and apparent ***YS*** than WPD scaffolds. ***E***_***c***_ for WPA scaffolds were found in range between 2.5 (WPA80) to 8.1 GPa (WPA50) whereas the value for WPD scaffolds ranged from 1.8 (WPD80) to 4.5 GPa (WPD50). The lowest ***E***_***c***_ value was measured for the reference model WP90 (1.0 GPa). Similar to ***E***_***c***_ WPA specimens demonstrated greater apparent ***YS*** than the WPD scaffolds. The greatest and the least ***YS*** were found to be 457.1 and 23.8 MPa, attributed to WPA50 and WP90 scaffold models, respectively shown in Fig 5C. The stress-strain curves for WPA50 and WPA60 showed a marked increase. This is attributed to the scaffold’s size, which is approximately 2.5× times smaller than the reference scaffold, and printing inaccuracy as the reduced size complicates the fabrication and post fabrication cleaning processes. For instance, the unit cells of WPA50 were quite small (1.12×1.12×1.12 mm^3^), making it challenging to remove the un-melted metal powder from the inner voids of the scaffolds through their tiny apertures during post-processing. This residual powder added extra mass when weighing the scaffold, directly affecting its relative density. An increase of 0.075 grams in mass due to retained powders resulted in a 0.2 unit rise in relative density. Thus, the discrepancy was mainly due to ineffective post-processing.

### 3.2 Structural analysis using FE method

Under the applied axial displacement, the equivalent (von-Mises) stress distributions on the scaffolds are shown in Fig 4B at 25% compression. The stress is distributed more on the longitudinal struts than on the lateral struts. Moreover, scaffold models of the same porosity from both groups show identical stress distributions. Fig 5B shows the compressive stress curves plotted against strain (%). The early linear nature of the stress-strain curves followed by the non-linear evolution qualitatively matched the characteristic curves obtained under ductility tests. Calculated ***E***_***c***_, and ***YS*** of scaffold models are shown in Fig 5C. ***E***_***c***_ and ***YS*** increase as the relative density increases. Further, they were found to be nearly equal for the scaffolds with similar relative density from both the study groups. The compression results showed that the ***E***_***c***_ for WPA scaffolds were found to range from 2.6 to 14.6 GPa and for WPD scaffolds, the values ranged from 2.2 to 13.4 GPa. The lowest ***E***_***c***_ value was predicted for the reference model WP90 (0.9 GPa). The ***E***_***c***_ and apparent ***YS*** results indicate that the WPA specimens have greater strength than the WPD models. The greatest and the least ***YS*** i.e. 306 MPa and 21.2 MPa, are attributed to WPA50 and WP90 scaffold models, respectively. For all corresponding scaffold model, FE results were found to be slightly on the higher side compared to the experimental results, the discrepancy between obtained results are 11% (WP90), 3.8% (WPA80), 7.8% (WPA70), 28.9% (WPA60), 44.1% (WPA50), 18.1% (WPD80), 42.2% (WPD70), 56.2% (WPD60), 66.4% (WPD50), respectively.

### 3.3 Comparison of mechanical properties

Fig 5D presents relative modulus versus relative density plots of WP scaffolds corresponding to both FE analyses and the in vitro experiments. The FE and experimental results of WPA and WPD scaffolds were well fitted having coefficient of determination (*R*^*2*^) of 0.99. Values of coefficient ***C*** and exponent ***n*** are also shown in the Fig 5D. In summary, the FE results of WP structures had ***n*** values ~2, suggesting a bending or twisting controlled mode of deformation. This suggests that the scaffolds are flexible and behaving less like a brittle material. Contrarily, the experimental results exhibited lower ***n*** values ~1, indicating, the material behaves linearly in response to compressive loads, similar to a linear elastic material.

### 3.4 SN curve

Fig 5E presents the S-N curves for the designed as well as fabricated scaffolds. The fatigue strength coefficients and exponents were theoretically calculated using Eqs 10–16, based on the stress generated in the scaffolds during the quasi-static compression tests in both experimental and FE analysis. The plotted curves encompass a range where *N*_*F*_≥1, ensuring that all stress combinations (*σ*_*m*_ and *σ*_*a*_) on or below the Soderberg line are deemed safe for design purposes. The curves indicate that as the *σ*_*a*_ decreases, the finite life of the scaffold increases, illustrating the typical inverse relationship between applied stress and the number of cycles to failure [70]. The figure provides a direct comparison of the fatigue life between the experimentally fabricated scaffolds and the corresponding FEA predictions, showing a generally close agreement thus validating the accuracy of the FE model. The calculated *σ*′_*f*_ for each scaffold was included in the figure legend, corresponding to the respective scaffold model. A notable trend is observed where increasing scaffold relative density leads to a significant increase in *σ*′_*f*_ indicating that more porous scaffolds exhibit lower fatigue resistance and vice a verse, which is critical for optimizing scaffold design for specific load-bearing applications.

The WPA scaffold group demonstrates superior fatigue strength compared to the WPD scaffolds among the fabricated samples. Specifically, WPA70 exhibits the highest fatigue strength of 32.4 MPa at 10^4^ cycles, which gradually decreases to 20.9 MPa at 10^6^ cycles. Furthermore, WPD50 shows a fatigue strength of 29.9 MPa at 10^4^ cycles, decreasing to approximately 19.3 MPa at 10^6^ cycles. However, WP90 has the lowest fatigue strength, ranging from 5 to 3.2 MPa for 10^4^ −10^6^ cycles, respectively, positioning them closer to cancellous bone in terms of fatigue performance. The fatigue strength curves for WPA50 and WPA60 were not shown in the figure because the finite *S*_*ys*_ for these scaffolds could not be determined in the stress strain curves due to their smaller size.

The FE analysis results align well with the experimental data, particularly for WP90, WPD80, WPD70, WPA80, and WPA70, indicating the accuracy of the FE models in predicting fatigue behaviour. However, the FE analysis tends to slightly overestimate the fatigue strength, especially for scaffolds with higher ρ*/***ρ***_**0**_, likely due to idealized assumptions in the model that do not fully capture the complexities of real-world fatigue behaviour.

### 3.5 Hemodynamic analyses

**Fig 6:**
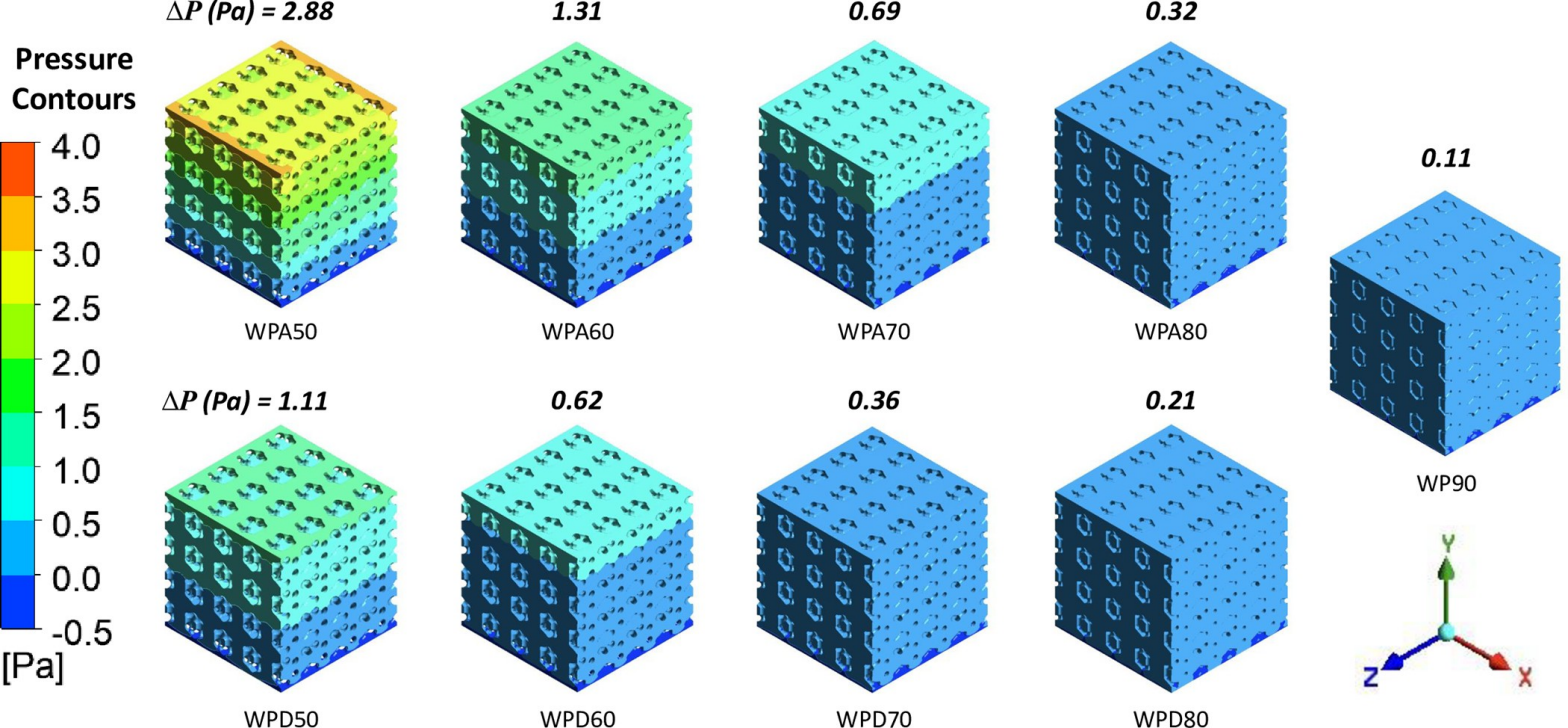
Pressure distribution contours for the WP scaffolds.

Fig 6 depicts the pressure distribution within the three-dimensional structure of the scaffold’s porous domain in the contour format. The maximum pressure appeared at the inlet and progressively reduced to zero at the outlet validating the boundary conditions. Average pressure drops, ***ΔP*** responses are shown in Fig 6. As the ρ*/***ρ***_**0**_ of the scaffold increases, the ***ΔP*** across the scaffolds was found to be increasing. However, WPA scaffolds predicted greater ***ΔP*** than the WPD scaffolds, almost twice for identical porosity. The greatest and the least pressure drops were observed for WPA50 (2.88 Pa) and WP90 (0.11 Pa) scaffolds, respectively.

**Fig 7:**
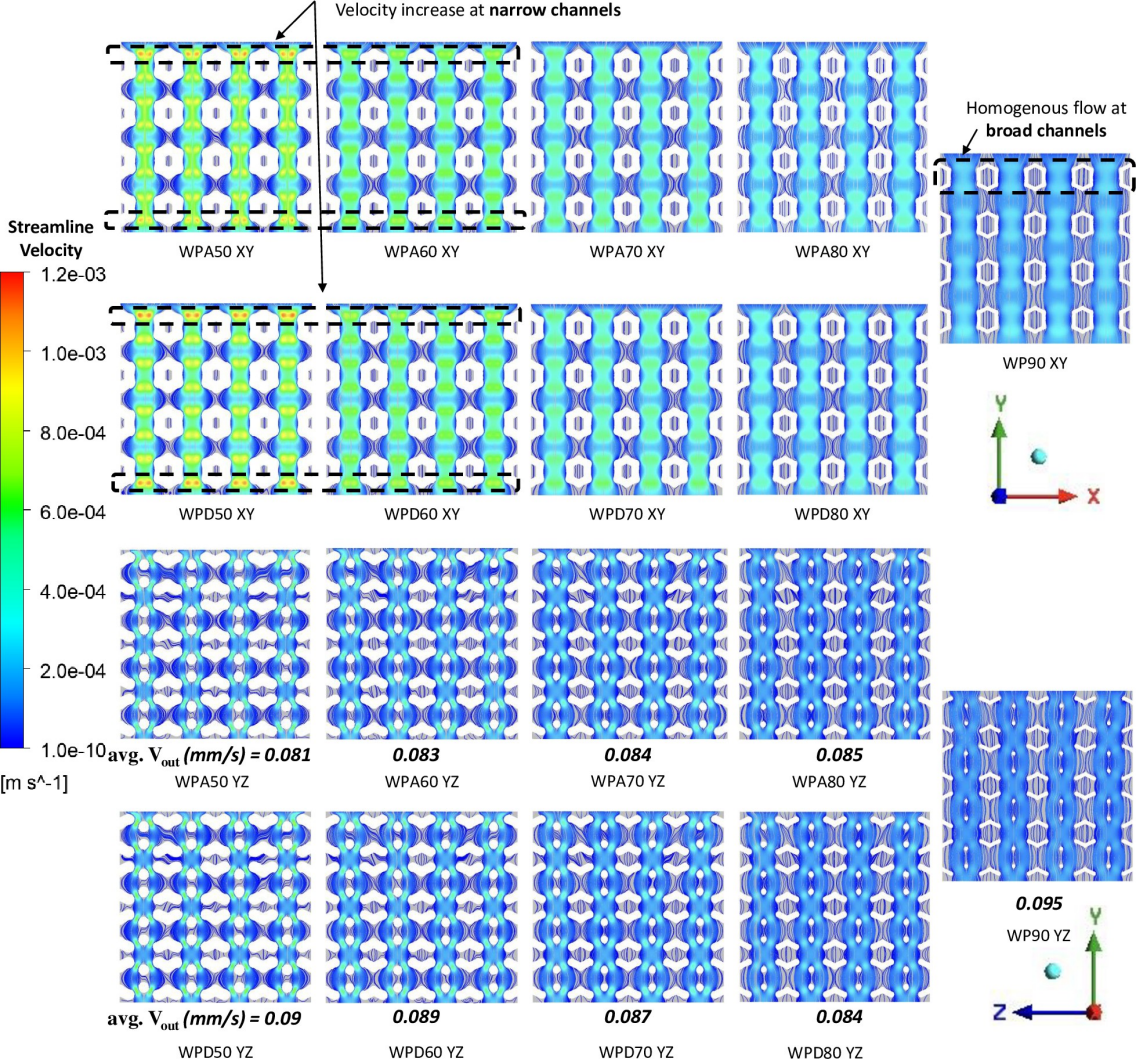
Velocity contours for different scaffold models on mid *x-y* plane and *y-z* plane.

Blood flow velocity contours in two cross sectional midplanes, i.e. ***x-y*** and ***y-z*** planes, are shown in Fig 7. The initial velocity of all scaffolds was set to ***Vi*** = 0.1×10^−3^ mm/s, which experienced an escalation as the fluid was passing through the interconnected pores in the structure. Notably, the maximum flow velocities were observed at the most constricted passages and necks within all scaffold’s designs. For instance, WPA50 and WPD50 scaffolds exhibit relatively smaller throats and narrower internal channels relative to other WP models with higher porosity, resulting in velocities as high as 1.16×10^−3^ mm/s. Furthermore, scaffolds characterized by 80–90% porosity were found to exhibit a uniform velocity distribution across their passages.

**Fig 8:**
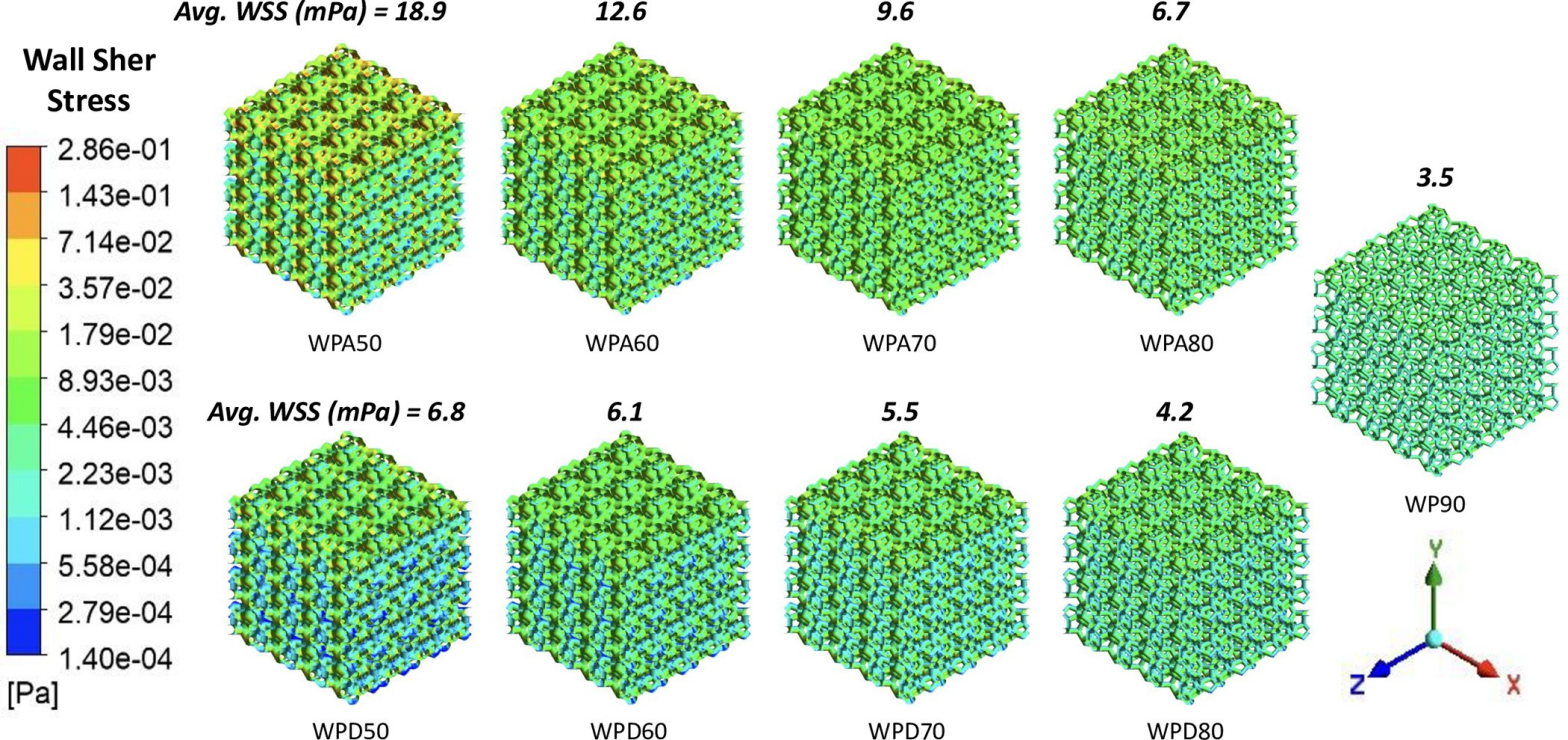
Wall shear stress (*WSS*) contours for different WP scaffolds.

The ***WSS*** contours on the scaffolds’ internal walls and average ***WSS*** values within all models are shown in Fig 8. The average ***WSS*** results agree with the relative density, as the values were found to be increasing as the scaffold’s relative density increases. Scaffolds with identical porosity show similar ***WSS*** contours. The highest ***WSS*** was seen to take place in the constricted areas of the channels within the scaffolds, a result of increase flow velocities. Consequently, uniform distribution of ***WSS*** was predicted on the scaffolds with relatively higher porosity (70%, 80% and 90%) as compared to less porous (50% and 60%) scaffolds.

**Fig 9:**
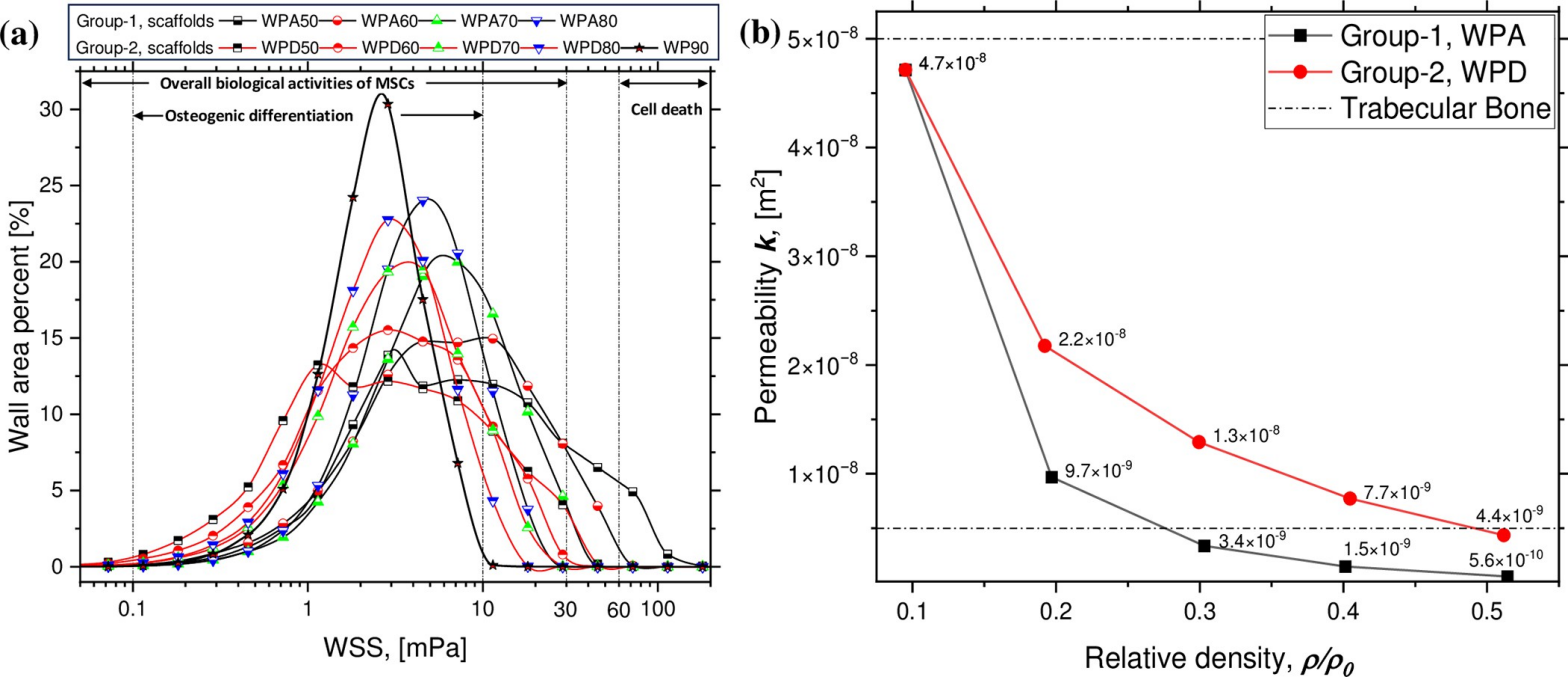
WSS and permeability: (a) frequency curves of wall shear stress distribution in different WP scaffold groups on its percentage wall area; (b) permeability curves plotted against relative density of Group-1 and Group-2 scaffolds compared with actual trabecular bone’s permeability range.

Fig 9A and 9B show the ***WSS*** distribution frequency over the scaffold’s surface area and predicted permeability of the scaffold models, respectively. WPA scaffolds possessed higher ***WSS*** values than WPD scaffolds. Consequently, ***WSS*** distribution frequency curves of WPA scaffolds shifted towards the apoptosis stimulus range. In contrast, WPD scaffold’s ***WSS*** values were distributed over the stimulus range that supports biological activity. The ***WSS*** distribution range of WPA and WPD scaffolds are 0.3–113.8 mPa and 0.3–45.4 mPa, respectively. Moreover, calculated permeability values for WPD scaffolds were substantially greater compared to the WPA specimens. As the ρ*/***ρ***_**0**_ of the scaffold increased, the permeability values decreased. Among all the scaffold models, WP90 predicted the highest permeability (4.71×10^−8^ m^2^) while the lowest permeability value was predicted for the WPA50 model (5.6×10^−10^ m^2^).

## 4. Discussion

This study aims to address the limitations of existing metal scaffolds used for bone implants, which often exhibit a higher modulus than bone and may not align with the biological requirements of bone applications. We conducted a comprehensive structural and hemodynamic analysis of nine distinct scaffold designs, all featuring the same WP architecture yet varying in feature dimensions. Our analysis encompassed a range of factors, including mechanical properties, compressive modulus, yield strength, pressure drop, blood flow pattern, wall shear stress, and permeability. The findings of our investigation, presented herein, asses the efficacy of these scaffold designs, and explore potential of these novel scaffolds as viable alternatives to natural trabecular bone in orthopaedic applications.

FE predicted stress-strain curves demonstrated relatively higher ***Ec*** and ***YS*** as compared to the experimental outcomes. Similar findings were reported in matching studies [73–75], where nearly all simulated ***Ec*** were found to be greater than the experimental outcomes, showcasing a maximum difference of ~23%, ~52% and ~45% respectively, between the corresponding measured and the predicted values. A plausible explanation for this could be the geometrical inconsistencies and microstructural deviations produced during fabrication process, which led to the significant decline in mechanical properties, such as reduction in ***Ec*** by approx. 10% to 20% [76] and a decrease in ***YS*** by up to almost half of the FE predictions [77]. Additionally, the non-uniform heat accumulation and quenching procedure likely had resulted in microstructural defects, which might have affected the actual structural properties of fabricated samples, causing discrepancy between the experimental and simulated results. As depicted in Fig 4A, the erosion of solid struts, i.e. material fracture might have occurred in the printed WP scaffolds. When the compression strain reached *ε* = 5%, the material failure was found predominantly in the diagonal sections of vertical struts and the junctions of inclined struts owing to the bending deformation and tensile failure, respectively. However, minimal element erosion was observed in less porous samples, aligning with the observation that the WPD sample exhibited more ductility than WPA scaffolds. It can thus be confidently stated that the ductility of WP scaffolds went up with the increase in relative density.

Fig 5D presents a comparison of the mechanical properties derived from our experiments and their corresponding FE validation results. We used the G-A model and the Carter and Hays trabecular bone model as references. This comparison allowed us to understand the deformation mechanisms unique to each group of scaffolds. However, during comparison it was noted that in WPA scaffolds (i.e. WPA50-WPA80) the increase in the relative densities was attributed to the un-melted, non-removable powder, which did not contribute as an integrated solid structural part to influence the mechanical performance of the scaffolds under axial compression load. To negate the effect of the added mass, the trend from the fitted curve of the obtained results was used to compare the mechanical properties of the respective scaffold groups. Thus, the difference in density should not significantly impact the comparison of mechanical properties between different groups. In fact, the error may proportionately influence as the relative density increases. Thereafter, FE results of both scaffold groups corroborate the G-A model trend and are positioned in a zone between the G-A and trabecular bone model. Although the fabricated scaffold’s compression trend is consistent with the FE results, the measured values are lower than the FE results and do not overlap (Fig 5D). Therefore, experimental results have been compared with previously reported explicit FE analysis on WP lattices [46]. As the curves of the experimental results matches with the trend in the reported explicit analysis results, it is evident that experimental results can be validated based on Johnson-Cook (JC) material model.

Experimental results of the scaffolds with the relative density of around 0.3 and below from both study groups coincides with the behaviour of trabecular bone. This suggests that scaffolds of WP architecture having porosity 70% can be best suited to replace trabecular bone, while scaffolds with much higher relative density (>0.3) can be employed to match the modulus of cortical bone.

The G-A model has been traditionally used for assessment of open cellular structures and metallic foams [15, 77–79]. This power-law equation offers a classification system for lattice structures and cellular solids, distinguishing them as either stretch-dominated or bending-dominated on the basis of their mechanical properties. Its relevance to additively manufactured or 3D printed porous specimens, however, has been challenged as significant discrepancies have been noted vis-à-vis experimental findings. This is largely attributed to the substantial variations between CAD designs and the final geometry of samples manufactured using DMP, particularly when dealing with smaller features. These variations influence both the manufacturability and the mechanical characteristics of the final product [41, 79]. A recent investigation has underscored underlying inconsistencies between fabricated lattice specimens and the G-A model, especially in terms of distortion patterns [41]. The G-A model is designed to analyse deformation in porous material through either stretching or bending, which are singular modes of deformation. It excludes the possibility of combined deformation modes like bending coupled with stretching and shearing. The model’s underlying assumption is that each strut within the lattice structure should be elongated and slim (i.e. slender beam), conforming to the Euler-Bernoulli beam theory. However, such model is not suitable for non-slender struts typically seen in 3D printed lattice structures (where ***l/d<5***), because such designs undergo a multi-mode deformation process [41].

The *N*_*F*_ is an important parameter to define safe operating range for orthopedic implants. Implants with *N*_*F*_≥1 are considered safe [68–70]. The fatigue results indicate that the 3D-printed Ti6Al4V scaffolds, particularly other than WP90, have fatigue characteristics that can be finely tuned to match those of human bone, depending on the specific application. The close alignment of WPA70, WPD50 and WPD60 scaffolds with the fatigue strength of cortical bone [72] suggests that these designs are well-suited for load-bearing orthopaedic applications where cortical bone replacement or augmentation is required. The slightly lower fatigue strength of WPD80, WPD70 and WPA80 scaffolds, on the other hand, aligns more closely with cancellous bone [71], making them more suitable for applications where lower mechanical loads are expected, such as in bone grafts or spinal cages. The validation of experimental results with FE analysis provides confidence in the use of computational models to predict the long-term performance of these scaffolds in clinical settings. The slight overestimation by the FE models at higher cycles could be attributed to differences in surface roughness, microstructural variations, or other imperfections in the actual 3D-printed scaffolds that are not fully accounted for in the models. This suggests that while FE analysis is a powerful tool for preliminary design and optimization, experimental validation remains crucial for ensuring the reliability of orthopaedic implants.

The ***WSS*** in the hemodynamic analysis (Fig 9A) serves as a mechanical stimulus for MSCs. When ***WSS*** is within the range of 0.5–30 mPa, it encourages MSCs to deposit mineralized extracellular matrix (ECM) within 3D scaffolds. However, ***WSS*** levels exceeding 60 mPa are harmful and may lead to cell death [30]. The ***WSS*** distribution for WPA and WPD scaffolds has been compared to the optimal ***WSS*** stimulus range. Analysis indicates that the likelihood of apoptosis induced by either group of scaffolds is minimal. Both groups are capable, to a certain degree, of prompting MSCs to deposit mineralized ECM in 3D scaffolds. Excluding the outer edge, the internal ***WSS*** for each scaffold group is predominantly within the range indicated by the dashed line. The final output of the hemodynamic analysis involves comparing permeability (***k***) of various scaffold groups with that of trabecular bone, where 0.5 < ***k*** (10^−8^ m^2^) < 5 [16–20]. The permeability values corresponding to WPA50, WPA60, WPA70 and WPD50 scaffolds are lower than the acceptable limit within the human body. However, the permeability of WPD60, WPD70, WPD80, WPA80 and WP90 scaffolds were found to lie within the range of trabecular bone. Scaffolds with a relative density around 0.3 or lower show a higher degree of match, particularly those in the WPD group. Despite having the same relative density, the permeability of the WPA scaffolds was found to be lesser. This was perhaps due to the shorter strut length of the WPA scaffolds compared to that of the WPD scaffolds, resulting in a smaller aperture that disrupts fluid flow more frequently. The Table 5 shades information regarding proposed applicability of the studied WP scaffolds, considering the varying bone properties across different anatomical regions.

**Table 5:** Proposed applications of WP scaffolds in bone replacement across different anatomical regions.

Bone Properties	Example	Scaffolds
High Flow Rate, High Strength	Femur, Tibia, Pelvic, Spinal bones	WPD60, WPD50
Low Flow Rate, High Strength	Skull Bones, Diaphysis of Long Bones	WPA60, WPA50
Low Flow Rate, Low Strength	Ribs, Clavicle, Scapula, Phalanges	WPA80, WPA70
High Flow Rate, Low Strength	Sternum, Vertebrae, Carpels, Tarsals	WPD80, WPD70

## 5. Conclusion

In this study, we assessed the newly proposed WP scaffolds (Ti-alloy) as a substitute to lattice scaffolds for bone tissue engineering. As such, FE structural analysis, CFD analysis and uniaxial compression tests were performed to comprehensively assess the structural, hemodynamic and morphometric properties of two distinct groups of WP scaffolds.

The primary findings of our study are as follows:

- Designed scaffolds were fabricated successfully employing DMP additive manufacturing technique. WPD scaffolds exhibited less printing error than WPA scaffolds and were found to be more consistent.
- WPA scaffolds exhibited slightly superior compressive modulus as compared to WPD scaffolds (22%– 63% depending on the porosity).
- The mechanical performances of fabricated WP scaffolds of 70%, 80% 90% porous group (relative density 0.3 and lower) were found ideal in comparison with natural trabecular bone tissues.
- WPD scaffolds showed improved hemodynamic properties as ***WSS*** and permeability values were found to be within the range of trabecular bone, i.e. 0.01–45.36 mPa and 0.77×10^−8^–4.7×10^−8^ m^2^, respectively, thus implying enhanced osteogenic potential. It may be noted here that average and maximum wall shear stress of scaffolds have significant influence on cell growth, MSC differentiation and bone mineralization.
- WPA70, WPD50 and WPD60 were found to be most promising as cortical bone replacement while WPD80, WPD70 and WPA80 scaffolds align more closely with trabecular bone due to their comparable fatigue strength.

By highlighting the lack of extensive investigation on WP scaffolds for bone replacement, this study introduces novel scaffold design aimed at improving bone replacement substitutes and includes a detailed hemodynamic analysis to predict blood flow through the scaffolds. It also explores the fatigue life of WP scaffolds, discusses the shortcomings of the Gibson-Ashby (GA) model while comparing mechanical properties of 3D printed porous structures, and uses the Basquin’s equation of fatigue strength for both cortical and cancellous bone to allow designers to map novel scaffolds in terms of fatigue life matching those of the bone. This altogether contributes to the development of more effective bone replacement materials.

In summary, the engineered WP Ti-scaffolds have not only met the necessary load-bearing requirements for bone replacement, but also have shown excellent morphometric and blood flow characteristics. The overall preclinical metrics highlight the potential for improved osseointegration, better structural conformities and more opportunities for patient specific customization with enhanced manufacturability.

Future endeavours in this field will involve carrying out in vitro studies to evaluate the potential for cells attachment and proliferation of WP scaffolds. Additionally, the scaffolds will be tested under combined loading conditions, such as compression, bending and torsion, as well as physiological loading conditions, to evaluate their performance and survivability when implanted in bone. Efforts will also focus on enhancing the fabrication quality of WP scaffolds to improve their surface integration with host tissue. These improvements will aid in the ongoing refinement and enhancement of WP scaffolds, further affirming their potential for use as bioengineered substitute in orthopaedic applications.

## Supporting information

S1 Data (XLSX)
